# Modeling Climate Impacts on Tree Growth to Assess Tree Vulnerability to Drought During Forest Dieback

**DOI:** 10.3389/fpls.2021.672855

**Published:** 2021-08-26

**Authors:** Cristina Valeriano, Antonio Gazol, Michele Colangelo, Ester González de Andrés, J. Julio Camarero

**Affiliations:** ^1^Instituto Pirenaico de Ecología (IPE-CSIC), Zaragoza, Spain; ^2^Departamento de Sistemas Naturales e Historia Forestal, Universidad Politécnica de Madrid, Madrid, Spain; ^3^School of Agricultural, Forest, Food and Environmental Sciences, University of Basilicata, Potenza, Italy

**Keywords:** climate warming, dendroecology, die-off, growth decline, process-based growth model, *Pinus pinaster*, tree rings

## Abstract

Forest dieback because of drought is a global phenomenon threatening particular tree populations. Particularly vulnerable stands are usually located in climatically stressing locations such as xeric sites subjected to seasonal drought. These tree populations show a pronounced loss of vitality, growth decline, and high mortality in response to extreme climate events such as heat waves and droughts. However, dieback events do not uniformly affect stands, with some trees showing higher symptoms of drought vulnerability than other neighboring conspecifics. In this study, we investigated if trees showing different vulnerabilities to dieback showed lower growth rates (Grs) and higher sensitivities to the climate in the past using dendroecology and the Vaganov-Shashkin (VS) process-based growth model. We studied two *Pinus pinaster* stands with contrasting Grs showing recent dieback in the Iberian System, north-eastern Spain. We compared coexisting declining (D) and non-declining (ND) trees with crown defoliation values above and below the 50% threshold, respectively. The mean growth rate was lower in D than in ND trees in the two stands. The two vigor classes showed a growth divergence prior to the dieback onset and different responsiveness to climate. The ND trees were more responsive to changes in spring water balance and soil moisture than D trees, indicating a loss of growth responsiveness to the climate in stressed trees. Such an interaction between water availability and vigor was reflected by the VS-model simulations, which provided evidence for the observation that growth was mainly limited by low soil moisture in both sites. Such an interaction between water availability and vigor was reflected by the VS-model simulations, which provided evidence for the observation that growth was mainly limited by low soil moisture in both sites. The presented comparisons indicated different stand vulnerabilities to drought contingent on-site conditions. Further research should investigate the role played by environmental conditions and individual features such as access to soil water or hydraulic traits and implement them in process-based growth models to better forecast dieback.

## Introduction

In the last decades, accelerated climate warming has caused a reduction in soil moisture, thereby exacerbating drought stress (Trenberth et al., 2014). Warmer and drier conditions have led to hotter droughts, which negatively impact forests worldwide and subsequently cause forest dieback (Allen et al., 2010, 2015). Such a loss in tree vigor is characterized by crown defoliation and increased mortality rates (Carnicer et al., 2011; Camarero et al., 2015a). Therefore, there is a deep concern for how the forecasted climate warming can expand forest dieback events, thus leading to a weakening of the terrestrial carbon sink and reducing the ability of forests to mitigate climate change (Anderegg et al., 2013). Climate-driven forest dieback is expected to increase in extent and severity in climate-change hotspots such as the Mediterranean Basin region, where seasonal changes in water availability limit tree growth and forest productivity (Vicente-Serrano et al., 2014; Gazol et al., 2018). In this region, climate models have forecasted increases in the frequency and intensity of hotter droughts during the 21st century (Giorgi and Lionello, 2008).

A better understanding of forest dieback processes is needed to identify their climatic causes and ascertain why some individuals are more vulnerable to drought stress than other conspecific, neighboring individuals (McDowell et al., 2008, 2011). Specifically, individual vulnerability may be related to site differences (e.g., soil depth) or intrinsic tree traits such as growth rates (Grs) (Pedersen, 1998). Different studies have found low Grs preceding dieback or tree death (Cailleret et al., 2017) while trying to extract early-warning signals of those radial-growth series (Camarero et al., 2015a; Cailleret et al., 2019). In conifers, declining (D) trees often showed low Grs prior to tree death, and this was associated with hydraulic failure caused by drought and xylem embolism (Choat et al., 2012, 2018; Adams et al., 2017). These results suggest that vulnerability to drought depends on “legacy effects” due to the cumulative impacts of climate stress on tree growth and vigor (Anderegg et al., 2015; Kannenberg et al., 2020).

Long-term, radial growth data allowed the reconstruction of how trees react to successive droughts and respond to such through changes in vigor expressed by differential leaf shedding rates, growth reduction, or tree death (Dobbertin, 2005). Thus, in this study, we compared the Grs and responses to climate variability of trees showing different needle shedding patterns. To delve into the climatic constraints of radial growth dynamics, we used the process-based Vaganov-Shashkin (VS) growth model (VS model hereafter, Vaganov et al., 2006). We argued that mechanistic rather than correlative approaches based on growth models will allow a better understanding of drought-induced dieback and the mortality process (Hendrik and Cailleret, 2017). This mechanistic model determines daily radial Grs as a function of daily climatic conditions by explicitly accounting for non-linear relationships between climate and growth (Tychkov et al., 2019). Relatively simple simulation frameworks such as those provided by the VS model allow the understanding of the major climatic constraints of growth, which is a key question in dieback processes (Sánchez-Salguero et al., 2017, 2020).

In this study, we focused on *Pinus pinaster*, a Mediterranean pine species which exhibits strong variability in response to drought throughout its distribution range in the Western Mediterranean Basin related to local climate conditions and provenance variability (Bogino and Bravo, 2008; Vieira et al., 2009, 2013; Sánchez-Salguero et al., 2018). We hypothesized that: (i) declining (hereafter D) trees will show lower Grs than non- declining (hereafter ND) trees prior to the dieback onset, (ii) D trees will show a higher long-term vulnerability to drought than ND trees, i.e., the radial growth of D trees will be more negatively impacted by dry and warm conditions during the growing season, and (iii) the more pronounced sensitivity of growth to drought stress in D trees, inferred by using the VS model, will explain its preferential dieback.

## Materials and Methods

### Study Site

**FIGURE 1:**
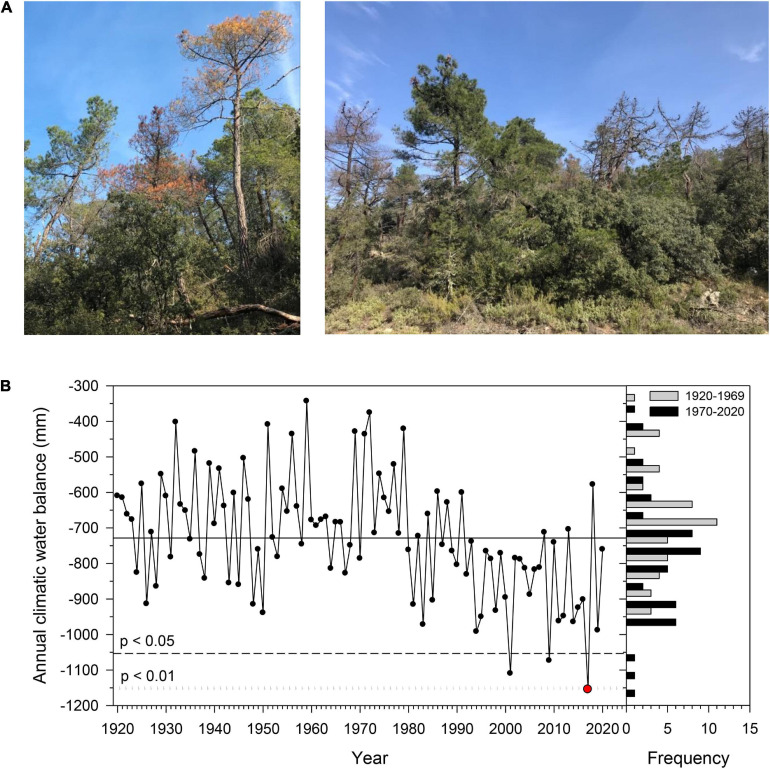
**(A)** Views of non-declining (ND), declining (D), dying, and recently dead *Pinus pinaster* trees sampled in the Miedes study site. **(B)** Temporal variability of the annual climatic water balance (P-PET) according to the data from the Daroca meteorological station. The dashed and dotted lines show the threshold for considering water balances significantly lower than the long-term mean (period 1920–2020) at the 0.05 and 0.01 significance levels, respectively. The histograms show the distribution of the water balance for the compared 1920–1969 and 1970–2020 periods. The red symbol highlights the 2017 drought.

Two different forests (Orera, Miedes) were studied in the central Iberian System, Aragón, north-eastern Spain (Table 1). These are two natural *P. pinaster* Ait. Stands that were subjected to light thinning in the past and now show recent canopy dieback and elevated mortality rates (after 2017), which may account for 22–35% of trees in some places (Figure 1A). The Orera site is located in steeper slopes (slope range 15–20°) than the Miedes site (slope range 0–5°). The understory of these forests is formed by *Quercus ilex* L., *Cistus laurifolius* L., and *Arctostaphylos uva-ursi* (L.) Spreng. The climatic conditions are the continental Mediterranean with low precipitation (Prec.) and strong temperature contrasts. The average annual temperature is 12°C, and the annual Prec. is 423 mm with a peak in spring and a secondary maximum in autumn. The period with a water deficit starts in June and may last until October (Supplementary Figure 1). The lithology of the zone is dominated by quartzites producing acid, rocky soils with sandy-loamy texture, and being relatively shallow (20–50 cm).

**TABLE 1:** Main features of the study sites and sampled trees.

Site	Latitude N	Longitude W	Elevation (m)	Tree type	Dbh (cm)	Age at 1.3 m (years)	Defoliation (%)
Orera	41.31	1.45	884	ND	36.0 ± 1.7b	82 ± 3	4.9 ± 2.2a
				D	31.5 ± 1.2a	78 ± 3	94.8 ± 2.2b
Miedes	41.27	1.43	961	ND	26.1 ± 0.9b	92 ± 2	10.7 ± 1.7a
				D	21.3 ± 0.7a	88 ± 2	58.0 ± 3.1b

*“ND” and “D” are non-declining and declining trees, respectively. Values are means ± SE. Different letters indicate significant (p < 0.05) differences between vigor classes within each site according to Mann–Whitney tests.*

### Sampling and Growth Chronologies Building

Sampling was done in 2019 and 2020. We measured the diameter at 1.3 m (Dbh, diameter at breast height) and visually assessed the percent of crown defoliation in pairs of neighboring, dominant trees with different defoliations but similar Dbh (Table 1). We considered two groups of vigor based on crown defoliation following Camarero et al. (2015a): D trees with more than 50% of defoliation and ND trees with less than 50% defoliation (refer examples in Figure 1A).

We took two cores per tree at 1.3 m using Pressler increment borers (Haglöf Sweden, Sweden). In total, 67 trees were sampled (Table 2). The collected cores were air-dried and carefully sanded to distinguish the rings following standard procedures in dendrochronology (Fritts, 1976). Samples were then visually cross-dated, and tree-ring widths (RW) were measured with a 0.001 resolution using scanned images (resolution 2400 dpi) and the CDendro software (Larsson and Larsson, 2018). Tree age at 1.3 m was estimated by counting the number of rings in the cores with pith or with curved inner rings. We also counted latewood intra-annual density fluctuations (IADFs) by inspecting the cores under a binocular microscope (Leyca, Wetzlar, Germany). The visual cross-dating was checked using the software COFECHA (Holmes, 1983).

**TABLE 2:** Tree-ring width (RW) data and statistics, first-order autocorrelation (AR1), mean sensitivity (MS), and correlation with mean series of D and ND trees in the Orera and Miedes study sites.

Site	Tree type	No. trees	No. radii	Tree ring width (mm)	AR1	MS	Correlation with mean series
Orera	ND	14	26	1.15 ± 0.08	0.75 ± 0.02	0.40 ± 0.01a	0.82 ± 0.01b
	D	23	46	1.08 ± 0.06	0.69 ± 0.03	0.42 ± 0.01b	0.79 ± 0.01a
Miedes	ND	15	30	0.85 ± 0.04b	0.64 ± 0.03a	0.43 ± 0.01a	0.81 ± 0.01b
	D	15	30	0.73 ± 0.03a	0.74 ± 0.01b	0.46 ± 0.01b	0.73 ± 0.01a

*“ND” and “D” are non-declining and declining trees, respectively. Values are means ± SE for the common period 1930–2019. Different letters indicate significant (p < 0.05) differences between vigor classes according to Mann–Whitney tests.*

The RW series were standardized and detrended. First, Friedman’s super smoother functions with intermediate smooth values were fitted to individual RW series to obtain ring-width indices (RWI) that preserved high-frequency variability. Second, we removed most first-order autocorrelations by fitting autoregressive models. Third, we obtained the mean series of pre-whitened RWIs or chronologies using bi-weight robust means. These mean series were calculated for D and ND trees in the two study sites separately. These analyses were performed using the package dplR (Bunn, 2008) in the R statistical package (R Core Team, 2020). We calculated the Expressed Population Signal (EPS) and the mean interseries correlation (rbar) to assess the coherence and replication of the resulting series (Wigley et al., 1984). We considered the period 1930–2019 with EPS > 0.85 to be well replicated. The rbar was calculated for 20-year intervals that shifted every year.

### Climate Data

Climate data were obtained at daily and monthly resolution from the Daroca meteorological station (41°06′54″N, 1°25′0″W, 782 m a.s.l.), which is located 18 km away from the sampling sites. The data series consisted of daily mean temperature (Tm), maximum temperature (Tx), minimum temperature (Tn), and Prec. for the period 1920–2020. Additionally, seasonal Tm and total Prec. were calculated for winter (December to February), spring (March to May), summer (June to August), and autumn (September to November). We also obtained 1.25°-gridded, monthly values of 1-m soil moisture for the period 1970–2016 from the European Centre for Medium-Range Weather Forecasts (ECMWF) Reanalysis (ERA)-Interim reanalysis (Dee et al., 2011). In addition, we also calculated the climatic water balance or difference between Prec. and potential evapotranspiration (P–PET). The PET was calculated using the FAO-56 Penman-Monteith equation (Allen et al., 1998).

### VS Growth Model

We focused on inter-annual Grs and calibrated and validated observed series of RW indices by comparing them with simulated series (Tumajer et al., 2017). The VS model (VS-oscilloscope ver.1.362) was used for modeling the Miedes and Orera RWI series of D and ND trees. The VS model is a process-based growth model of intermediate complexity that has been widely applied and validated in conifers (refer, among others, Vaganov et al., 2006; Touchan et al., 2012; Shishov et al., 2016; He et al., 2017; Popkova et al., 2018; Tychkov et al., 2019; Tumajer et al., 2021). The model simulates daily Grs focusing on xylogenesis (enlargement, division, and differentiation of tracheids) and considering the data daily values of air temperature, Prec., and radiation as input. The model parameters defined the integrated Gr and the relative GRs due to soil moisture (Gr_W_) or temperature limitations (Gr_T_). During the year, days were classified according to the main climatic limitations of growth as: temperature – (Gr_T_ < Gr_W_) or moisture-limited (Gr_W_ < Gr_T_) and optimal (Gr_M_ = Gr_T_ = 1).

We used data from daily climate variables (air temperature, Prec., radiation) and the standard RWI series as input data. In total, 18 parameters were used to calibrate the model (refer a description of the model and parameters in Supplementary Table 1). The study period was 1930−2019, and it was divided into two sub-periods (1930−1969 and 1970−2019) to calibrate and verify the model predictions, respectively (Cook and Kairiukstis, 1990). The model was adjusted by modifying the parameters until the correlation between the four observed and predicted RWI series reached maximum values. The degree of adjustment between the observed and predicted RWI series was assessed using Pearson correlation coefficients (*r*) and the root mean squared error (Tychkov et al., 2019).

### Statistical Analyses

All analyses were performed in R (R Core Team, 2020). We evaluated the trends of the daily and seasonal climatic variables (Tx, Tn, Prec. and P–PET) with the Mann–Kendall test (τ). Comparisons between years (climate data), variables of sites, or vigor classes (defoliation) were assessed using Mann–Whitney tests.

To assess the differences in growth variability between the two vigor groups, pointer years (Cropper, 1979) and resilience components (Lloret et al., 2011) were calculated using the R package PointRes (van der Maaten-Theunissen et al., 2015). Positive and negative pointer years were calculated considering a 7-year window size, a 0.75 growth deviation, and a minimum percentage of 75% of trees displaying positive or negative event years. Then, the resilience components proposed by Lloret et al. (2011) were calculated using the standard RWI series. We selected the four most severe droughts occurring after the wet-cool 1970s (1983, 2001, 2009, and 2015) because the annual water balance was below the mean in most years of the 1980−2020 period (Figure 1B). We considered a 3-year window for pre- and post-disturbance (drought) periods. The resistance index was the ratio between RWIs during drought and the 3-year previous period, and the recovery index was the ratio between the 3-year post-drought period and the drought RWI values. Finally, the resilience index was the ratio between the 3-year RWI values after and before the drought. We compared the individual values of the three resilience indices (resistance, recovery, and resilience) between ND and D trees using Mann–Whitney tests.

Climate-growth relationships were assessed by calculating bootstrapped Pearson correlations between monthly climatic parameters and the mean residual RWI series for the period 1930–2019. Monthly Tx, Tn, and water balance from the previous September to the current September were included in the windows of analysis. To assess the changes through time in climate-growth associations we also calculated the moving climate-growth relationships for selected climate variables considering the 30-year moving intervals that shifted every year from 1930 to 2019. The calculations were performed for ND and D trees using the R package Treeclim (Zang and Biondi, 2015).

We fitted generalized additive mixed models (GAMMs; Wood, 2017) to study the temporal trends in simulated daily Grs using vigor class and date (DOY, day of year) as explanatory variables. The year was used as a random factor, and a first-order autocorrelation structure (AR1) was introduced to account for temporal autocorrelations within each year. Separate analyses were also performed for the two sub-periods (1930−1969 and 1970−2019). Finally, we fitted GAMMs considering the interaction between the spring (March, April, and May) water balance (P − PET) and DOY to test if the variation in Gr differed between years with different water availabilities. An AR1 was again used to account for temporal autocorrelations. The GAMMs were fitted using the mgcv package (Wood, 2011), and the visreg package (Breheny and Burchett, 2017) was used to visualize regression graphs.

## Results

### Seasonal Climate Trends and Variability

The mean climatic water balance for the period 1920–2020 was −728 mm with a SD of 167 mm. This variable has shown a negative and significant trend during that period (τ = −0.31, *p* < 0.001) with a mean rate of change of −2.44 mm year^–1^ (Supplementary Table 2). The 2017 annual water balance (−1,155 mm) was significantly lower than the long-term mean at the 0.01 significance level due to very low spring (−274 mm) and autumn water balances (−343 mm) (Figure 1B and Supplementary Figure 2). These two low values correspond to the minimum values of the long-term record for their respective seasons (standardized anomalies of −1.9 and −2.29 in spring and autumn, respectively). The mean Txs of the four seasons significantly increased during the last 100 years, especially in summer (τ = −0.41, *p* < 0.001; Supplementary Figure 3).

### Growth, Defoliation, and Resilience Indices

The diameter breast height at 1.3 m (Dbh) of D trees was smaller than ND in both sites (Table 2). In Miedes, the RW was also significantly lower in D than in ND trees, despite both vigor classes showing similar ages. However, ND trees showed higher first-order autocorrelation and mean sensitivity than D trees in the same site. The ND trees showed a higher mean correlation with the mean RW series than D trees in both study sites (Table 2). The mean defoliation rate of sampled trees was significantly lower (Mann–Whitney *U* = 389, *p* = 0.022) in Miedes (mean ± SE, 34.3 ± 4.7%) than in Orera (61.7 ± 7.3%). We observed very high (>90%) defoliation rates in 6.7 and 47.4% of trees sampled in Miedes and Orera, respectively, albeit the growth rate was lower in Miedes.

**FIGURE 2:**
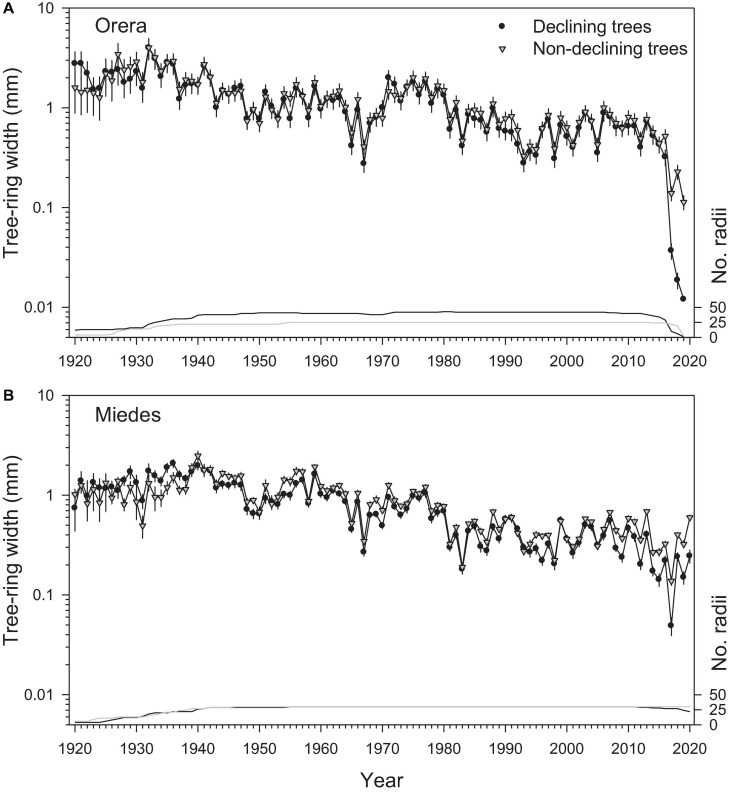
Long-term radial-growth patterns of D (black circles) and ND (gray triangles) trees in the **(A)** Orera and **(B)** Miedes study sites. Values are means SE (note the logarithmic scales in the left *Y*-axis). The number of measured radii (lines) is shown in the right *Y*-axis.

The long-term pattern of radial growth variability was similar between D and ND trees (Figure 2 and Supplementary Figure 4). Within each site, both ND and D mean series of RWI were significantly correlated during the 1930 − 2019 period (Miedes, *r* = 0.79; Orera, *r* = 0.94; *p* < 0.001 in both cases). Similar strong associations were found between sites either for ND (*r* = 0.66) or D (*r* = 0.68) trees. The coherence in the year-to-year growth variability between sites and vigor classes suggests a similar response to climatic constraints such as water deficits.

We found a clear growth divergence between ND ad D trees in both sites due to a growth reduction of D trees after the 2017 severe drought and after 2008 (about 12 years before the dieback started) in Miedes (Figure 2). Trees from the Orera site also presented a reduction in the mean interseries correlation of D trees after the 1990s, which was subsequently reversed in the 2010s (Supplementary Figure 5).

The pointer years (Cropper values) were similar in magnitude and direction between D and ND trees (Supplementary Figure 6). The most negative pointer years coincided with dry years (e.g., 1983, 2001, 2009, 2015, and 2017). The latewood IADFs were usually observed during wet-cool years (e.g., 1952, 1961, and 1970s) with similar yearly frequencies in D and ND trees (Supplementary Figure 7). However, the D trees tended to form fewer IADFs after the 1980s as the climate warmed and dried and their Grs decreased.

The resilience indices were similar between D and ND trees in both sites apart from the 2001 recovery index with lower values in ND trees in the Orera site and the 2009 resistance index with higher values in ND trees from Miedes (Supplementary Figure 8).

In both sites, crown defoliation and recent growth rate (mean RW of the last formed 5 years) were inversely related (Supplementary Figure 9).

### Growth Responses to Climate: The Importance of Water Balance

Growth was enhanced by cool and wet conditions in May and June (Table 3). High water balance values from January to April and September were associated with higher Grs. In Miedes, high Tns from January to February improved growth. The growth responsiveness to climate was similar between ND and D trees apart from the positive influence of September water balance on the growth of ND trees in both sites. These relationships agree with the positive associations found between growth and spring to early summer soil moisture, which were stronger for ND than for D trees in Miedes (Supplementary Figure 10).

**TABLE 3:** Climate-growth relationships of D and ND trees in the Orera and Miedes sites.

Climate variable	Site	Tree type	Month												
			S^*t–1*^	O^*t–1*^	N^*t–1*^	D^*t–1*^	J	F	M	A	M	J	J	A	S
Mean maximum temperature (Tx)	Orera	ND	0.02	0.03	−0.04	0.13	−0.12	−0.11	−**0.22**	−0.07	−**0.29**	−**0.21**	−0.13	0.02	0.03
		D	0.05	0.01	−0.06	0.13	−**0.16**	−0.03	−0.12	−0.03	−**0.28**	−0.15	−0.09	−0.06	0.06
	Miedes	ND	−0.07	−0.02	−0.02	0.06	−0.07	−0.10	−**0.25**	−0.04	−**0.31**	−**0.35**	−0.15	−0.07	−0.02
		D	−0.08	−0.08	−0.06	0.06	−0.11	−0.03	−**0.27**	−0.05	−**0.31**	−**0.25**	−0.06	0.01	0.11
Mean minimum temperature (Tn)	Orera	ND	−0.08	−0.08	0.12	0.03	0.18	0.09	0.04	0.17	−**0.27**	−**0.20**	−0.09	−0.15	0.18
		D	−0.02	−0.08	0.15	0.05	0.13	0.11	0.06	0.15	−**0.27**	−0.12	−0.06	−0.14	0.16
	Miedes	ND	−0.09	−0.04	0.17	0.04	**0.20**	0.20	0.07	0.06	−**0.19**	−**0.33**	−0.08	−0.09	−0.04
		D	−0.06	−0.11	0.12	0.04	0.12	**0.19**	0.10	0.11	−**0.23**	−**0.29**	−0.02	−0.12	0.17
Water balance (P-PET)	Orera	ND	−0.06	−0.02	−0.01	0.13	**0.24**	**0.25**	**0.26**	**0.35**	**0.38**	**0.22**	0.21	0.11	**0.23**
		D	−0.08	0.05	0.06	0.15	**0.24**	**0.23**	**0.24**	**0.36**	**0.39**	0.17	0.16	0.15	0.18
	Miedes	ND	0.05	−0.01	−0.01	0.14	0.15	**0.26**	**0.31**	**0.25**	**0.45**	**0.32**	0.17	0.15	**0.20**
		D	0.08	−0.01	−0.01	0.10	0.09	**0.27**	**0.30**	**0.29**	**0.40**	**0.26**	0.12	0.05	0.14

*The values are Pearson correlation coefficients calculated by relating the mean series of ring-width indices (RWI) and monthly values of maximum and Tns with water balance. ND and D are non-declining and declining trees, respectively. Correlations were calculated from the prior (^t–1^) to the current September. Significant correlation coefficients are shown as bold values.*

**FIGURE 3:**
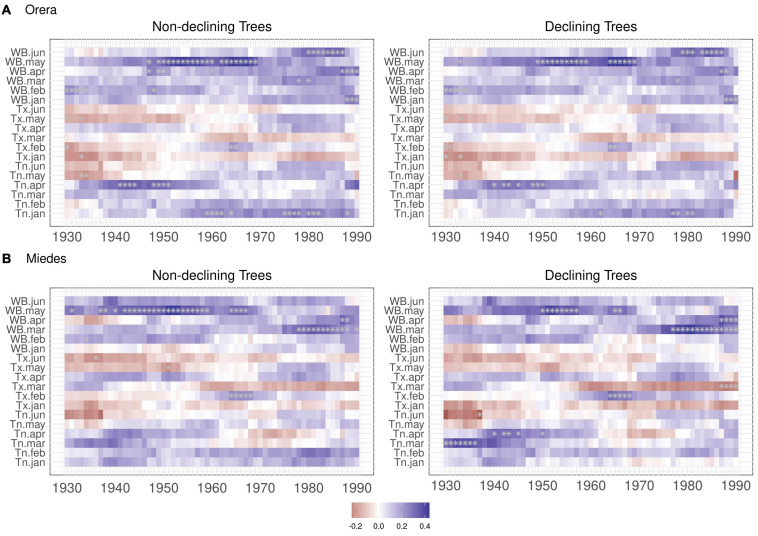
Moving climate-growth correlations of ND and D trees in the **(A)** Orera and **(B)** Miedes study sites. Plots show selected climate variables and their relationships with RWI considering 30-year moving intervals that shifted every year from 1930 to 2019. Values are plotted for the first year of each 30-year interval. Asterisks indicate significant (*p* < 0.05) correlations.

The moving climate-growth correlations showed that March–April water balance is gaining importance as a major driver of growth in Miedes. Despite this, the May water balance is losing relevance, whereas January–April and June water balances are becoming significant drivers of growth in Orera (Figure 3). These changes suggest shifts in the growing season, which we investigated using the VS model.

### Shifting Climatic Limitations of Tree Growth

**FIGURE 4:**
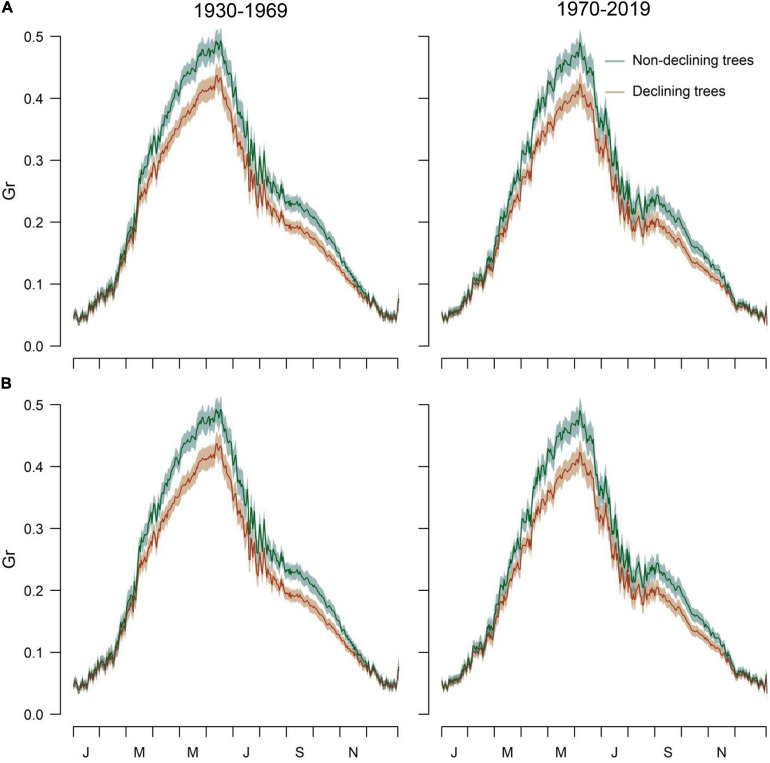
Simulated Grs of the VS model obtained by relating daily climate data and mean series of RWI of D and ND trees in the **(A)** Orera and **(B)** Miedes study sites considering the periods 1930–1969 and 1970–2019. The daily Gr values are means ± SD.

The simulated and observed RWI series of the calibration and verification periods were significantly correlated, indicating robust models (Supplementary Table 3 and Supplementary Figure 11). The Gr values peaked from early May to mid-June (Figure 4). A second autumn peak (September) was also observed, suggesting a facultative bimodal growth pattern. The GAMMs showed lower Grs of D than ND trees in both study sites and periods (Table 4 and Figure 4).

**TABLE 4:** Results of the generalized additive mixed models (GAMMs) fitted to study the simulated growth variability (Gr) in *P. pinaster* D and ND in two consecutive periods (1930−1969 and 1970−2019) in Orera and Miedes sites.

Site	Period	Tree type	Edf	*F*	*t*	*R* ^2^
Orera	1930−1969	ND	8.68	509.7**	10.35**	0.63
		D	8.57	385.1**		
	1970−2019	ND	8.72	456.7**	9.91**	0.66
		D	8.60	340.3**		
Miedes	1930−1969	ND	8.68	510.9**	10.41**	0.63
		D	8.57	385.1**		
	1970−2019	ND	8.72	457.4**	9.97**	0.59
		D	8.60	340.1**		

For each model, the degrees of freedom (Edf) and F values associated with the smooth parameter (DOY, day of the year) and their interaction with tree vigor (ND and D trees) are shown. In addition, the significance of the t statistic comparing vigor classes and the R^2^ of the model are presented. Significant values (P < 0.01) are indicated with **.

**FIGURE 5:**
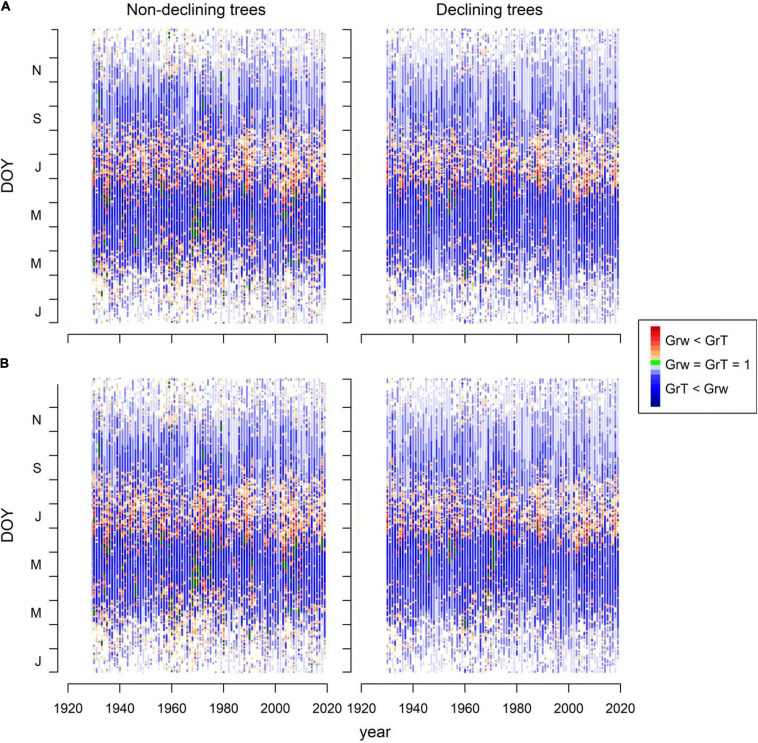
Simulated radial growth rates (Gr) based on the fits of the VS model to mean series of RWI of ND and D trees in the Orera **(A)** and Miedes **(B)** study sites. The graphs show the growth function (Gr) values for each day of the year (DOY, *Y*-axis) and considering the period 1930 – 2019 (*X*-axis). Plotted values are classified according to soil-moisture (Gr_W_ < Gr_T_, orange-red symbols) or temperature limitation of growth (Gr_T_ < Gr_W_, blue symbols). The green symbols indicate optimal growing conditions (Gr_T_ = Gr_W_ = 1).

**FIGURE 6:**
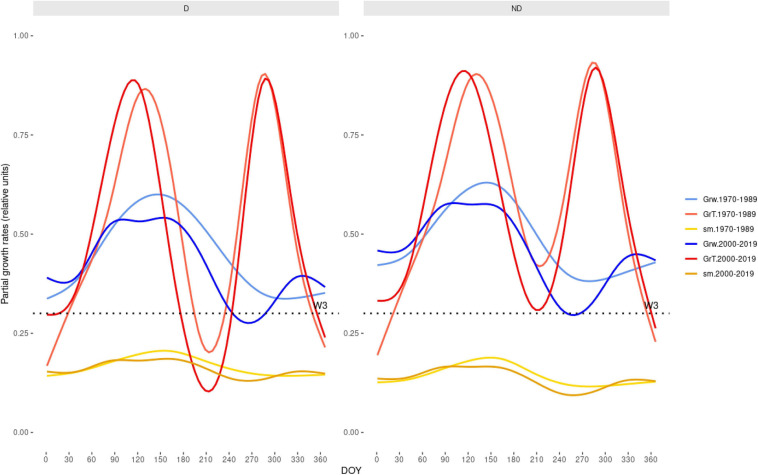
Tree growth is becoming more limited by warmer and drier climate conditions according to the Vaganov-Shashkin (VS) model simulations. The plot shows the partial Grs driven by soil moiture (GrW) or temperture (GrT) limitations of growth, and depending on soil moisture (sm) during the year (*X*-axis shows DOY). We compared wet-cool (1970–1989, mean annual water balance –673 mm) and dry-warm (2000–2019, mean annual water balance –873 mm) periods in the case of the trees from the Miedes site. The W3 is the soil moisture threshold needed for growth (refer Supplementary Table 1).

**FIGURE 7:**
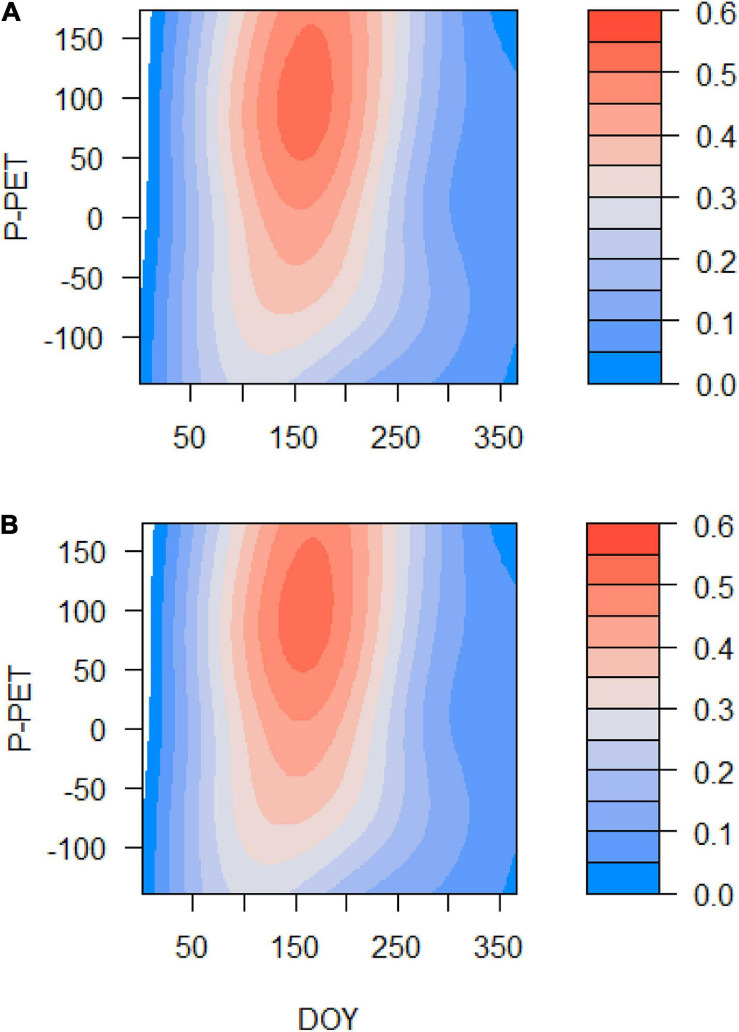
Effects of the DOY, the spring water balance (P – PET, *Y*-axis), and their interaction on the simulated Gr (color scale) in the Orera **(A)** and Miedes **(B)** study sites. The analyses correspond to GAMMs fitted to Gr data for the period 1930–2019.

Grs increased in years with higher spring water balance. We observed shifts in climatic limitations of growth with optimal conditions in wet-cool periods (e.g., the 1970s) and a trend toward higher importance of water balance and soil moisture as constraints of growth (Figure 5). This can be observed by comparing wet-cool with dry-warm periods in the most recent decades (the 2000s to 2010s), which shows simulated Grs below the soil moisture (sm) threshold for growth (W3, Figure 6). There was a significant interaction of spring water balance and DOY on Grs in both sites, indicating a shift in the climatic limitations of growth related to lower soil moisture availability due to a more negative water balance (Figures 1, 7 and Supplementary Table 4).

## Discussion

The findings contribute to recent research showing how successive and hotter droughts lead to pervasive growth reductions (Cailleret et al., 2017; Camarero et al., 2018) and trigger dieback and mortality in Mediterranean *P. pinaster* forests (Gea-Izquierdo et al., 2019; Férriz et al., 2021). The presented results confirm the first hypothesis that Grs of D trees were lower than ND trees in at least one site (Miedes), but refute the idea of D trees being more vulnerable to drought stress unlike what has been found in other conifer species such as *Abies alba* (Camarero et al., 2015a). We found that the growth of ND and D trees was severely impacted by warm and dry spring conditions and that climate is warming and drying in the study area, with a record spring hot drought found in 2017. In Miedes, ND trees were more responsive to water availability than D trees as confirmed in the correlation analyses with soil moisture. In Miedes, the growth of D trees is uncoupling from climatic constraints, perhaps because they are presenting legacy or carryover effects (Anderegg et al., 2015) of previous droughts, thus impairing their post-drought recovery.

The growth loss of D trees was recent in Orera, as it started in 2017, but could be dated back to 2008 in Miedes, where ND trees showed higher responsiveness to water availability. The findings are in line with previous studies showing that *P. pinaster* radial growth is very dependent on sufficient water availability and elevated soil moisture from the prior winter to early summer (Bogino and Bravo, 2008; Camarero et al., 2015b). The study species is considered a drought-avoiding conifer (Picon et al., 1996) with rapid stomatal closure in response to mild-water stress which, thereby, reduces photosynthetic rates (Ripullone et al., 2007). These responses could explain the elevated rates of needle shedding observed in D trees in this study and elsewhere (Férriz et al., 2021). Such irreversible canopy defoliation could reflect a hydraulic failure in many shoots and impair carbon uptake leading to dieback (McDowell et al., 2008).

The sensitivity of xylogenesis to inter- and intra-annual changes in water availability is also reflected in its ability to form abundant latewood IADFs and modulate its xylogenesis in seasonally dry sites (Vieira et al., 2009, 2010, 2013). However, this xylem plasticity has limits. This would explain how the extremely hot 2017 drought triggered the recent dieback process. The results suggest that ND trees from Miedes grew more than their D conspecifics and were more sensitive to spring water deficits, which agrees with a recent study (Férriz et al., 2021). The different impacts of drought on conspecific individuals could be caused by differential vulnerabilities to xylem embolism related to greater vulnerabilities to xylem conduit implosion and/or a lower ability to store carbohydrates or show post-drought recovery in D trees (Gaylord et al., 2015; Savi et al., 2020). In contrast, tree-ring data, VS model simulations, and IADFs show that ND and D trees from Orera were similarly stressed by drought. For instance, growth divergence between both vigor classes was only apparent after the severe 2017 drought. The correlation between the mean series of these classes was higher than in Miedes, suggesting a strong coherence in growth between conspecifics regardless of recent defoliation. In Orera, ND trees also produced a low frequency of IADFs since the 1980s, suggesting a drought-constrained or a low ability to show bimodal patterns. Interestingly, the VS model indicated a higher bimodality in ND trees, which responded more to the September Prec. This bimodality was probably more evident during wet-cool periods (the 1960s and 1970s) when growing conditions were less stressful and more IADFs were produced. This different response to climate suggests the higher growth plasticity of ND trees in response to autumn Prec., but this should be further investigated. Differences in growth responsiveness to climate between the two study sites cannot be attributed to genetic origin (cf. Sánchez-Salguero et al., 2018) since they are separated by ca. 5 km, and the two stands belong to the same provenance. Stand structure does not seem to explain these differences because, in both cases, we sampled open stands with low to mid densities (J. J. Camarero, personal observation) where competition for soil water was probably low. A potential factor explaining site-contingent responses or different vulnerabilities of neighboring trees may be related to the actual amount of water stored by soil, since Orera trees were growing on steeper slopes that may make them more stressed by warm and dry conditions. In contrast, D trees in Miedes could be growing on sites with shallow, rocky soils or showing intrinsic anatomic or physiological traits that reduce their drought resilience. We did not observe D, less vigorous trees showing significantly lower resilience to drought as has been observed for dying trees in many sites (DeSoto et al., 2020). However, only two vigor classes were considered in this study based on their contrasting crown defoliation. Further studies with larger samples allowing for a more complete classification of defoliation classes may be helpful to advance the understanding of the relationships between canopy defoliation and growth vigor in *P. pinaster*.

Process-based models have not been able to accurately predict drought-induced tree mortality (Hendrik and Cailleret, 2017; Choat et al., 2018), which limits the forecasting capacity of vegetation dynamics in a warmer and drier world. Physiological models explicitly consider hydraulic architecture and carbon allocation processes to simulate drought-induced dieback and tree mortality (Xu et al., 2013), but process-based models such as the VS model could be more easily parameterized to provide growth forecasts (Sánchez-Salguero et al., 2017; Sánchez-Salguero and Camarero, 2020). Such growth models could also be compared with retrospective analyses of hydraulic and carbon-use proxies obtained from RW, such as wood anatomical or isotope discrimination data (Gaylord et al., 2015; Pellizzari et al., 2016).

The modeling approach also has limitations, such as the relative simplicity of the VS model, which focused on carbon sinks (cambium activity) but did not consider physiological processes related to gas and water exchange and how they are linked to hydraulic failure (Plaut et al., 2012) as other more complex models do (Hendrik and Cailleret, 2017). The VS model simulates radial-Grs (RWI) with great accuracy but is not able to produce a series of absolute growth values (e.g., basal area increment), an output generated by other models dealing with carbon allocation (Li et al., 2014). Finally, the modeling framework uses input means and indexed growth series for D and ND trees. Despite this, more realistic approaches should deal with individual growth data to account for the huge growth variability among conspecific, coexisting trees. Such variabilities could be related to differences in soil features (e.g., depth, texture, and water holding capacity) or stand structure (e.g., tree-to-tree competition), which should be considered in further modeling exercises. Interestingly, some of the values used to parametrize the VS model, such as root depth, were realistic enough and produced reliable simulated Grs since *P. pinaster* forms most fine roots in the uppermost 40 cm of the soil in dry sites (Bakker et al., 2006).

## Conclusion

We studied recent dieback events in two *P. pinaster* stands using a retrospective approach and the process-based VS model. Dieback was characterized by recent growth declines and crown defoliation, which were responses to prior spring droughts. Overall, ND trees presented higher Grs. However, the growth responses to climate were also contingent on on-site conditions, with long and short growth declines prior to the dieback onset in Miedes and Orera sites, respectively. The growth of ND trees from the Miedes sites responded more to water balance and soil moisture, suggesting that D trees from this site were chronically stressed as inferred for all trees from the Orera site. Process-based growth models should be more widely used and refined to characterize the mechanisms of drought-induced dieback and be used as prospective tools to forecast forest dynamics under warmer and drier climate scenarios.

## Data Availability Statement

The original contributions presented in the study are included in the article/Supplementary Material, further inquiries can be directed to the corresponding author.

## Author Contributions

CV led the manuscript writing and the sample analyses with contributions from JC and AG. All authors participated in field sampling and data collection, and contributed to the writing of the manuscript and approved its final version.

## Conflict of Interest

The authors declare that the research was conducted in the absence of any commercial or financial relationships that could be construed as a potential conflict of interest.

## Publisher’s Note

All claims expressed in this article are solely those of the authors and do not necessarily represent those of their affiliated organizations, or those of the publisher, the editors and the reviewers. Any product that may be evaluated in this article, or claim that may be made by its manufacturer, is not guaranteed or endorsed by the publisher.
