# Supplementary material for: Modeling Climate Impacts on Tree Growth to Assess Tree Vulnerability to Drought During Forest Dieback
Source: Front Plant Sci. 2021 Aug 26;12:672855. doi: 10.3389/fpls.2021.672855 (PMC8426521; doi:10.3389/fpls.2021.672855)
Supplement: Supplementary file 1 [file Data_Sheet_1.docx]

Supplementary Material

**Supplementary Table S1.** Optimal model parameters estimated for the calibration of the VS model on *Pinus* pinaster chronologies over the period 1930–2019 in the Orera and Miedes study sites. Temperature is defined defined by four variables that describe the temperature regime of tree growth (minimum-T1, maximum-T4, range of optimal temperatures −T2 and T3). Two additional parameters defined the initiation of growth (Tbeg, accumulated temperature; tbeg, total days to achieve this temperature). Temperature factors are related to needle transpiration with two parameters (C2 and C3) characterizing the leaf resistance to water vapor diffusion and how this is impacted by air temperature. Precipitation is also considered through the calculation of the soil moisture parameters (minimum-W1, maximum-W4, range of optimal soil moistures −W2 and W3). Additional parameters were depth of root system, maximum of daily precipitation to saturate soil, fraction of precipitation penetrating soil, and the soil water capacity of drainage. Lastly, the model considers a minimum cambial cell growth rate (Vcr). Physiological and soil data were mainly obtained from field data and published literature.

| Description | Units | Parameter | Orera | | Miedes | |
| --- | --- | --- | --- | --- | --- | --- |
|  |  |  | D | ND | D | ND |
| Minimum temperature for tree growth | ◦ C | Tmin/T1 | 3 | 3 | 3 | 3 |
| Lower end of range of optimal temperatures | ◦ C | Topt 1/T2 | 10 | 11 | 10 | 11 |
| Upper end of range of optimal temperatures | ◦ C | Topt 2/T3 | 15 | 16 | 15 | 16 |
| Maximum temperature for tree growth | ◦ C | Tmax/T4 | 28 | 23 | 28 | 23 |
| Temperature sum for initiation of growth | ◦ C | Tbeg | 77 | 77 | 85 | 84 |
| The period of sum temp. to start growth | days | tbeg | 10 | 10 | 10 | 11 |
| Minimum soil moisture for tree growth | v/vs^1^ | Wmin/W1 | 0.01 | 0.01 | 0.01 | 0.07 |
| Lower end of range of optimal soil moistures | v/vs | Wopt 1/W2 | 0.27 | 0.27 | 0.27 | 0.27 |
| Upper end of range of optimal soil moistures | v/vs | Wopt 2/W3 | 0.30 | 0.30 | 0.30 | 0.30 |
| Maximum soil moisture for tree growth | v/vs | Wmax/W4 | 0.35 | 0.37 | 0.35 | 0.37 |
| Initial soil moisture | v/vs | W0 | 0.00 | 0.10 | 0.00 | 0.10 |
| Depth of root system | mm | lr | 350 | 350 | 300 | 300 |
| Maximum daily precipitation for saturated soil | mm/day | Pmax | 37 | 35 | 37 | 35 |
| Fraction of precipitation penetrating soil | rel. unit | C1 | 0.46 | 0.45 | 0.46 | 0.45 |
| First coefficient for calculation of transpiration | mm/day | C2 | 0.48 | 0.48 | 0.42 | 0.36 |
| Second coefficient for calculation of transpiration | mm/day | C3 | 0.11 | 0.08 | 0.05 | 0.05 |
| Coefficient for water drainage from soil | rel. unit | q | 0.01 | 0.01 | 0.01 | 0.01 |
| Minimum cambial cell growth rate | no units | Vcr | 0.05 | 0.05 | 0.04 | 0.04 |

^1^v/vs–relative volumetric soil moisture content.

**Supplementary Table S2.** Trends in seasonal climate variables (data from Daroca station) assessed for the period 1930–2019. Bold values are significant (*p* < 0.05).

| Climate variable | Season | Kendall tau | *p* |
| --- | --- | --- | --- |
| Mean maximum temperature | Winter | **0.294** | < 0.0001 |
|  | Spring | **0.271** | < 0.0001 |
|  | Summer | **0.407** | < 0.0001 |
|  | Autumn | **0.281** | < 0.0001 |
| Mean minimum temperature | Winter | 0.094 | 0.165 |
|  | Spring | 0.100 | 0.139 |
|  | Summer | **0.330** | < 0.0001 |
|  | Autumn | **0.196** | 0.004 |
| Precipitation | Winter | -0.106 | 0.117 |
|  | Spring | 0.009 | 0.890 |
|  | Summer | -0.089 | 0.187 |
|  | Autumn | -0.045 | 0.508 |
| Water balance (P−PET) | Winter | **-0.262** | 0.001 |
|  | Spring | -0.112 | 0.096 |
|  | Summer | **-0.265** | < 0.001 |
|  | Autumn | **-0.159** | 0.019 |

**Supplementary Table S3.** Results of the parametrization of the VS model fitted to chronologies of declining (D) and non-declining (ND) *Pinus pinaster* trees. The comparison between simulated and observed chronologies between calibration (1930−1969) and verification (1970−2019) periods are based on Pearson correlation coefficient (*r*) and root mean-squared error (RMSE). In all case these statistics were significant (*p* < 0.01).

| Site | Period | Tree type | *r* | RMSE |
| --- | --- | --- | --- | --- |
| Orera | 1930−1969 | ND | 0.63 | 0.209 |
|  |  | D | 0.68 | 0.258 |
|  | 1970−2019 | ND | 0.70 | 0.210 |
|  |  | D | 0.73 | 0.275 |
| Miedes | 1930−1969 | ND | 0.76 | 0.171 |
|  |  | D | 0.71 | 0.170 |
|  | 1970−2019 | ND | 0.62 | 0.257 |
|  |  | D | 0.63 | 0.262 |

**Supplementary Table S4.** Results of the Generalized Additive Mixed Models (GAMM) fitted to study the simulated growth rates in non-declining *Pinus pinaster* trees and their variation according to the spring climate water balance (P−PET, period 1930-2019) in Orera and Miedes study sites. For each model, the degrees of freedom (Edf) and *F* value associated to the smooth parameter (DOY) and their interaction with water balance are shown. In addition, the R^2^ values of the models are presented. Significance levels (*p* < 0.01) are indicated with **.

| Site | Edf | F | R^2^ |
| --- | --- | --- | --- |
| Orera | 19.76 | 292.0** | 0.62 |
| Miedes | 19.73 | 292.6** | 0.62 |

**Supplementary Figure S1.** Climate diagram and monthly water balance according to data from the Daroca meteorological station.

**Supplementary Figure S2.** Trends and variability in seasonal climatic variables related to (a) temperature and (b) precipitation and the climatic water balance (P−PET). Significant (p<0.05) trends are showing with lines of the same colors as climate variables. See also Table S2.

**Supplementary Figure S3.** Trends in daily maximum temperature anomalies and raw data observed during the months with maximum water deficit (July to September). Data correspond to the Daroca station, period 1920–2020. In the lower plot the black points show some outliers. Anomalies were calculated with respect to 1920–2020 averages.

**Supplementary Figure S4.** Residual, pre-whitened series of ring-width indices for the declining and non-declining trees in the two study sites.

**Supplementary Figure S5.** Moving correlations of the mean interseries correlation (rbar.eff) calculated among declining (filled symbols) and non-declining (empty symbols) individual series of ring-width indices trees in the (a) Orera and (b) Miedes study sites. Correlations were calculated using 20-year long periods and were corrected according to the change of sample size. Values are means ± SE.

**Supplementary Figure S6.** Pointer years in the declining and non-declining trees sampled in Orera (a) and Miedes (b) study sites. The bars are Cropper values (means ± SD) or normalized deviation of tree ring-width indices for 3-year windows.

**Supplementary Figure S7.** Frequency of latewood intra-annual density fluctuations observed in the latewood of declining and non-declining trees sampled in (a) Orera and (b) Miedes study sites


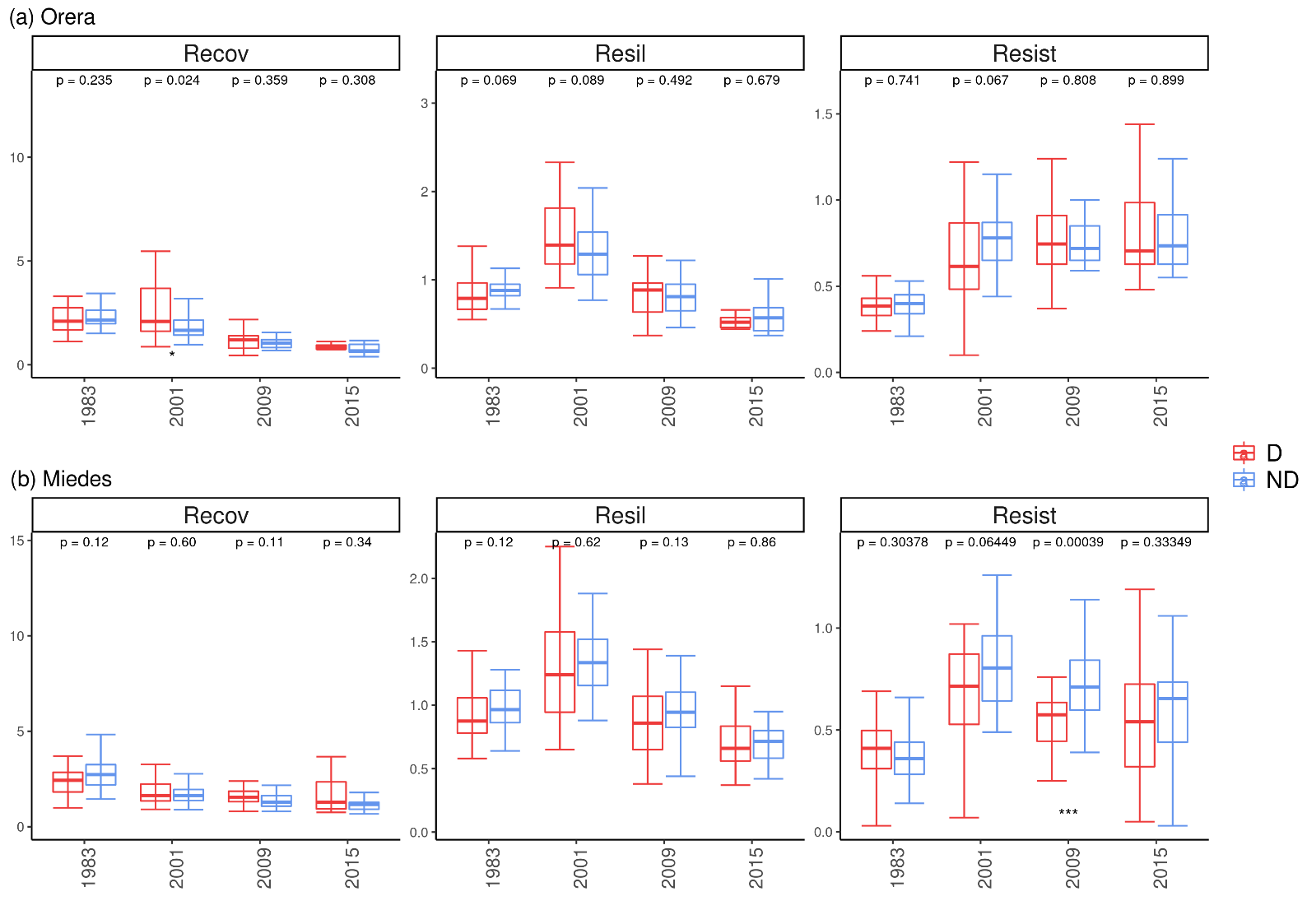


**Supplementary Figure S8.** Resilience growth indices (recovery, resilience and resistance) calculated considering selected droughts (1983, 2001, 2009 and 2015) for declining (D) and non-declining (ND) trees in the (a) Orera and (b) Miedes study sites and considering selected dry years (1983, 2001, 2009 and 2015). Significant differences between tree classes are indicated by asterisks (*, *p* < 0.05; ****p* < 0.001).

**Supplementary Figure S9.** Negative relationships between tree defoliation and recent growth rate in the two study sites (Orera, Miedes). The growth rate is the mean tree-ring width of the last formed five years (2015−2019 in Orera and 2016−2020 in Miedes). The statistics show Spearman correlation coefficients (*r_s_*) and associated significance levels (*p*).

**Supplementary Figure S10.** Correlations of estimated soil moisture (period 1970−2016, moisture estimated up to 1 m depth) and the mean series of ring-width indices of declining (black bars) and non-declining (grey bars) trees in the (a) Orera and (b) Miedes study sites. The dashed and dotted lines show the 0.05 and 0.01 significance levels, respectively. The abbreviations MAMJ are refers to mean soil moisture from March o June.

**
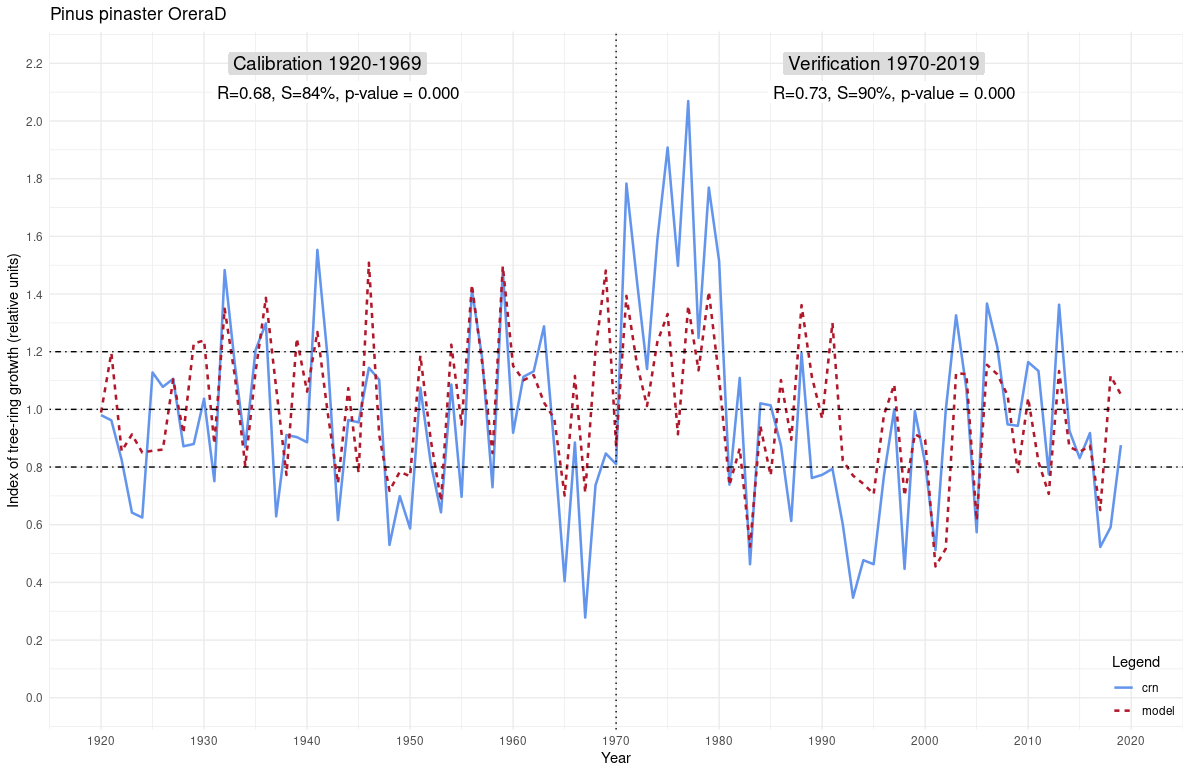
**

**
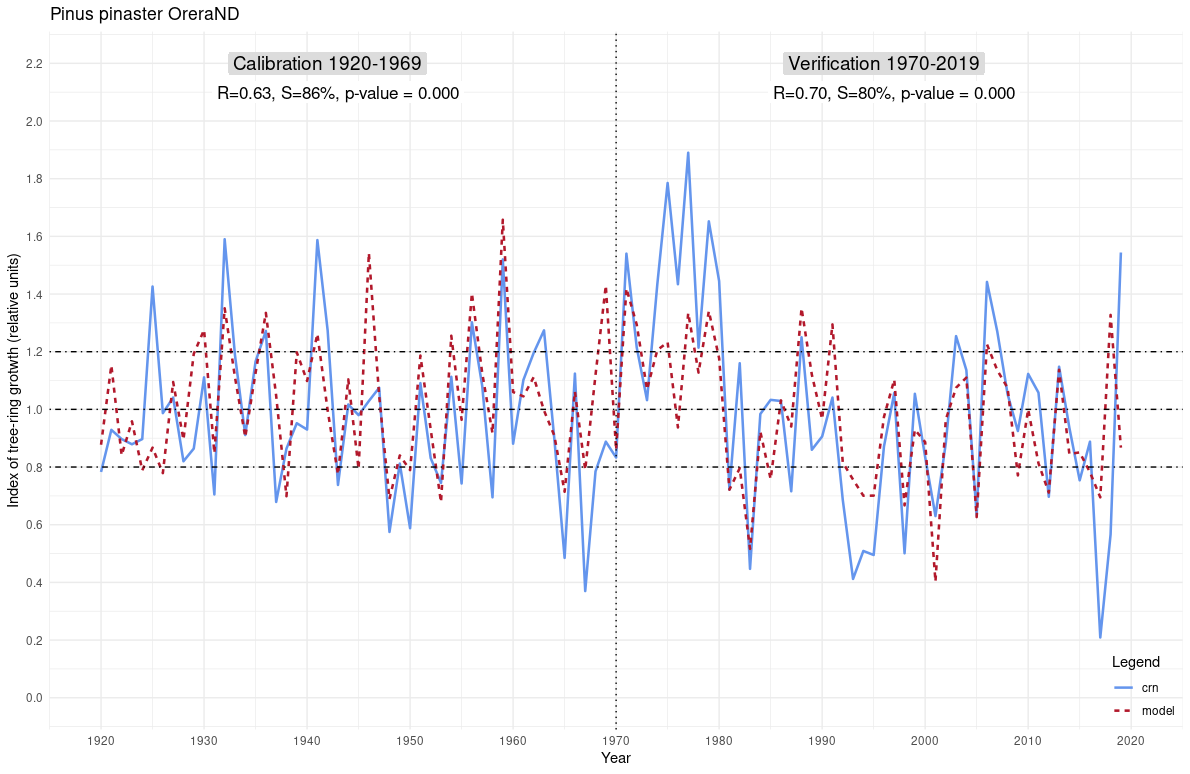
**

**
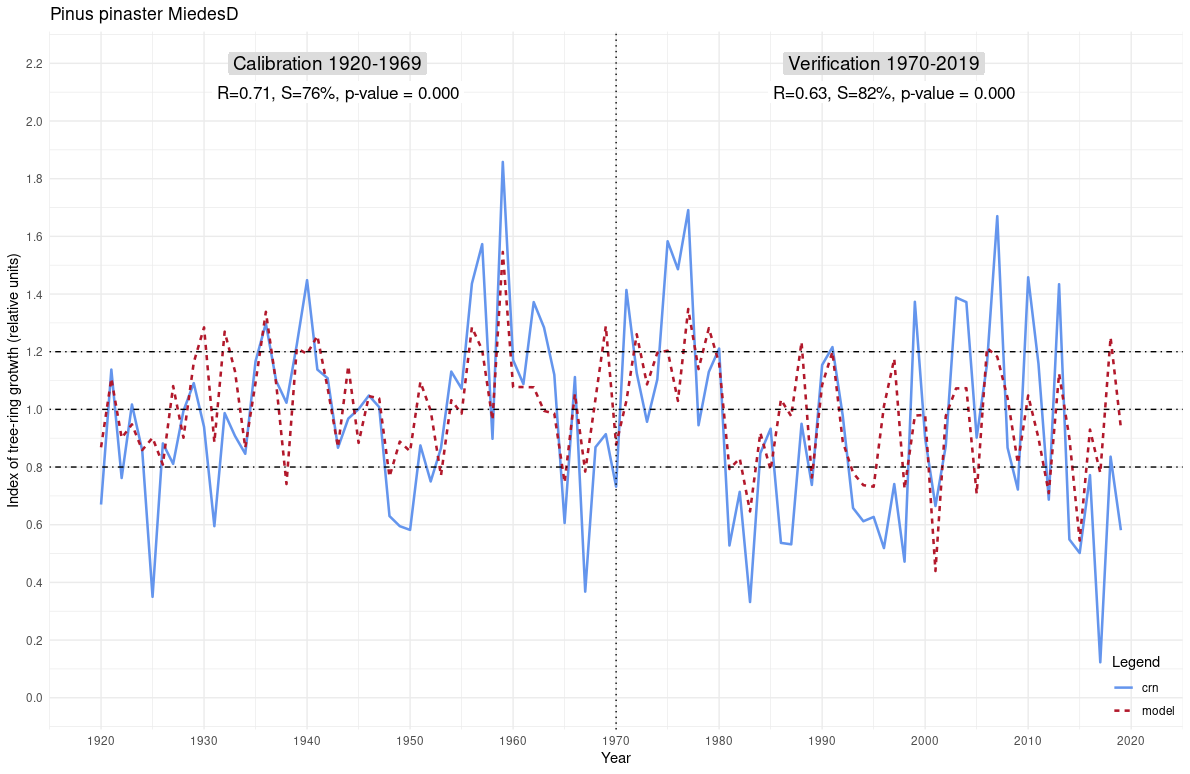

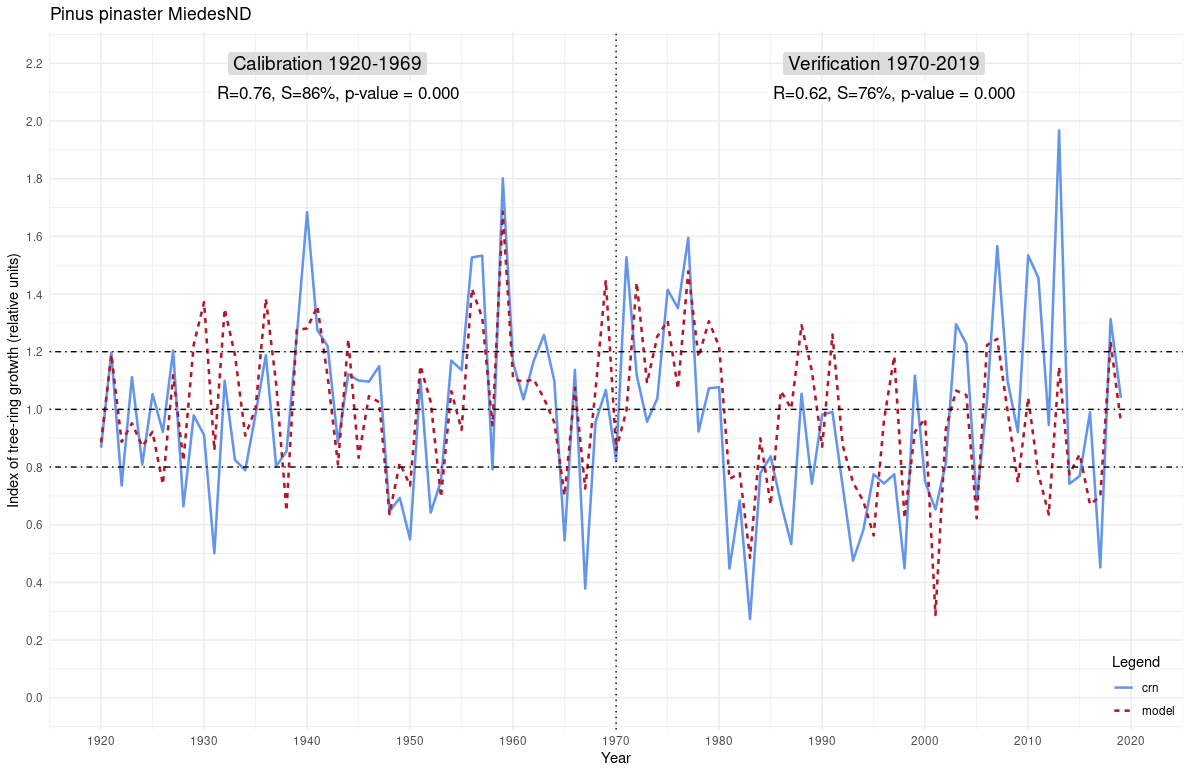
**

**Supplementary Figure S11.** Calibration-verification outputs of VS-model. Comparison of simulated (red dotted line) and observed standard (blue solid line) chronologies. The statistics means, R = Pearson correlation coefficient; S = Synchronicity index and the p-value of correlation (Table S3).
